# The targeted LHRH analog AEZS-108 alters expression of genes related to angiogenesis and development of metastasis in uveal melanoma

**DOI:** 10.18632/oncotarget.27431

**Published:** 2020-01-14

**Authors:** Klara Fodor, Nikoletta Dobos, Andrew Schally, Zita Steiber, Gabor Olah, Eva Sipos, Lorant Szekvolgyi, Gabor Halmos

**Affiliations:** ^1^University of Debrecen, Department of Biopharmacy, Debrecen, Hungary; ^2^Veterans Affairs Medical Center, Endocrine, Polypeptide and Cancer Insitute, Miami, FL, USA; ^3^University of Miami, Miller School of Medicine, Department of Pathology and Department of Medicine, Divisions of Oncology and Endocrinology, Sylvester Comprehensive Center, Miami, FL, USA; ^4^University of Debrecen, Department of Ophthalmology, Debrecen, Hungary; ^5^University of Debrecen, Faculty of Medicine, Department of Biochemistry and Molecular Biology, MTA-DE Momentum, Genome Architecture and Recombination Research Group, Debrecen, Hungary

**Keywords:** uveal melanoma, luteinizing hormone-releasing hormone (LHRH) receptor, angiogenesis, *MASPIN/SERPINB5*, AEZS-108 (AN-152/Zoptarelin Doxorubicin Acetate)

## Abstract

Uveal melanoma (UM) is the most common malignant tumor of the eye. Recently, we have established that 46% of UM specimens express LHRH receptors. This finding supports the idea of a LHRH receptor-targeted therapy of UM patients. Cytotoxic analog of LHRH, AEZS-108 exhibits effective anti-cancer activity in LHRH-receptor positive cancers. AEZS-108 is a hybrid molecule, composed of a synthetic peptide carrier and the cytotoxic doxorubicin (DOX). In the present study, we investigated AEZS-108 induced cytotoxicity and the altered mRNA expression profile of regulatory factors related to angiogenesis and metastasis in LHRH receptor positive OCM3 cells. Our results show that AEZS-108 upregulates the expression of *MASPIN/SERPINB5* tumor suppressor gene, which is downregulated in normal uvea and UM specimens independently from the LHRH receptor-ligand interaction. AEZS-108 also substantially downregulates hypoxia-inducible factor 1 alpha (HIF1A) expression. In order to investigate the mechanism of the induction of *MASPIN* by AEZS-108, OCM3 cells were treated with free DOX, D-Lys^6^ LHRH analog, or AEZS-108. qRT- PCR analysis revealed in OCM3 cells that AEZS-108 is a more potent inducer of *MASPIN* than free DOX. In conclusion, we show for the first time that AEZS-108 has a major impact in the regulation of angiogenesis thus plays a potential role in tumor suppression. Taken together, our results support the development of novel therapeutic strategies for UM focusing on LHRH receptors.

## INTRODUCTION

Although uveal melanoma (UM) is a rare disease, it is the most prevalent lethal ophthalmological tumor [1, 2]. Approximately 50% of the patients already manifests distant metastases, mostly in the liver at the time of the diagnosis [2]. In spite of the currently available systemic therapies, 90% of the patients with metastasis die within 1 year of the diagnosis of UM [3, 4]. Chemotherapy or partial hepatectomy only rarely prolongs the survival, emphasizing the necessity of developing more effective therapies [5].

The discovery of specific receptors for peptide hormones on cancer cells has led to the development of cytotoxic or radiolabeled hormone analogs that are appropriate for tumor localization and targeted therapy. Numerous preclinical studies have demonstrated the efficacy of chemotherapy based on cytotoxic peptide conjugates targeted to receptors on different tumors [6–10]. Clinical trials confirmed that targeted cytotoxic LHRH analog can improve the effectiveness of treatment and reduce general side effects [11–13].

Luteinizing Hormone-Releasing Hormone (LHRH) and its receptor (LHRH-R) are not limited to the hypothalamic-pituitary axis [14–18]. In the periphery, the LHRH system regulates gonadal functions and appears to serve as a growth factor of benign prostate hyperplasia [10, 19–21] and even in various cancers, including breast, lung, ovary, endometrial, urinary, colon, pancreas and prostate cancer [22–31].

In our previous study, we have revealed that more than 40% of human uveal melanomas express LHRH receptor type I [1]. AEZS-108 (formerly known as AN-152 / INN: Zoptarelin Doxorubicin Acetate) is a targeted cytotoxic LHRH-analog consisting of doxorubicin conjugated to D-Lys^6^ LHRH. AEZS-108 guides the chemotherapeutic agent specifically to those tumors that express LHRH-receptors, which could result in targeted cytotoxicity and less damage to healthy tissues [9, 11, 13, 30, 32]. AEZS-108 has been utilized in phase III clinical trials in advanced, recurrent or metastatic endometrial cancer, in phase I-II in castration resistant prostate cancer and ovarian cancer [9, 11, 13, 32]. Moreover, AEZS-108 was found to be able to inhibit the growth of doxorubicin resistant cells [31, 33].

In the present study our aim was to demonstrate the antitumor effects of AEZS-108 in a human uveal melanoma cell line. As we reported it previously OCM3 UM cell line express the receptor of LHRH localized on the cell membrane and in the cytoplasm, rendering them susceptible to AEZS-108 uptake [33, 34]. The detection of LHRH receptor in OCM3 cells has led to use AEZS-108 for targeted therapy of the tumor. Our results shows that AEZS-108, as well as doxorubicin significantly inhibited the proliferation of OCM3 human uveal melanoma cells. Angiogenesis has a pivotal role in the development of UM because lymphatic vessels are not present in the eye to promote distant metastasis [35, 36]. This special characteristics of UM also led us to investigate the effect of AEZS-108 on the expression of the genes involved in angiogenesis and metastasis in an *in vitro* model of UM. The qRT-PCR array results showed that AEZS-108 altered the expression of *MASPIN*, *HIF1A* and its target genes. Furthermore, qRT-PCR analysis revealed that AEZS-108 is a more potent inducer of *MASPIN* tumor suppressor than free DOX in OCM3 cells. *MASPIN* has been shown to be downregulated in normal uvea and UM tumor specimens.

Western blot analysis confirmed that the greatest changes of mRNA expression are at protein levels as well. To conclude, we report here, that OCM3 UM cell line expresses the LHRH receptor and LHRH rendering them susceptible to AEZS-108 uptake. AEZS-108 treatment resulted in changes in the expression of genes involved in the extracellular matrix (ECM) remodelling and angiogenesis. Our findings support the novel notion that AEZS-108 might be suitable as a potential drug candidate in targeted therapy of uveal melanoma.

## RESULTS

### OCM3 human uveal melanoma cell line expresses the human LHRH receptor type I

**Figure 1:**
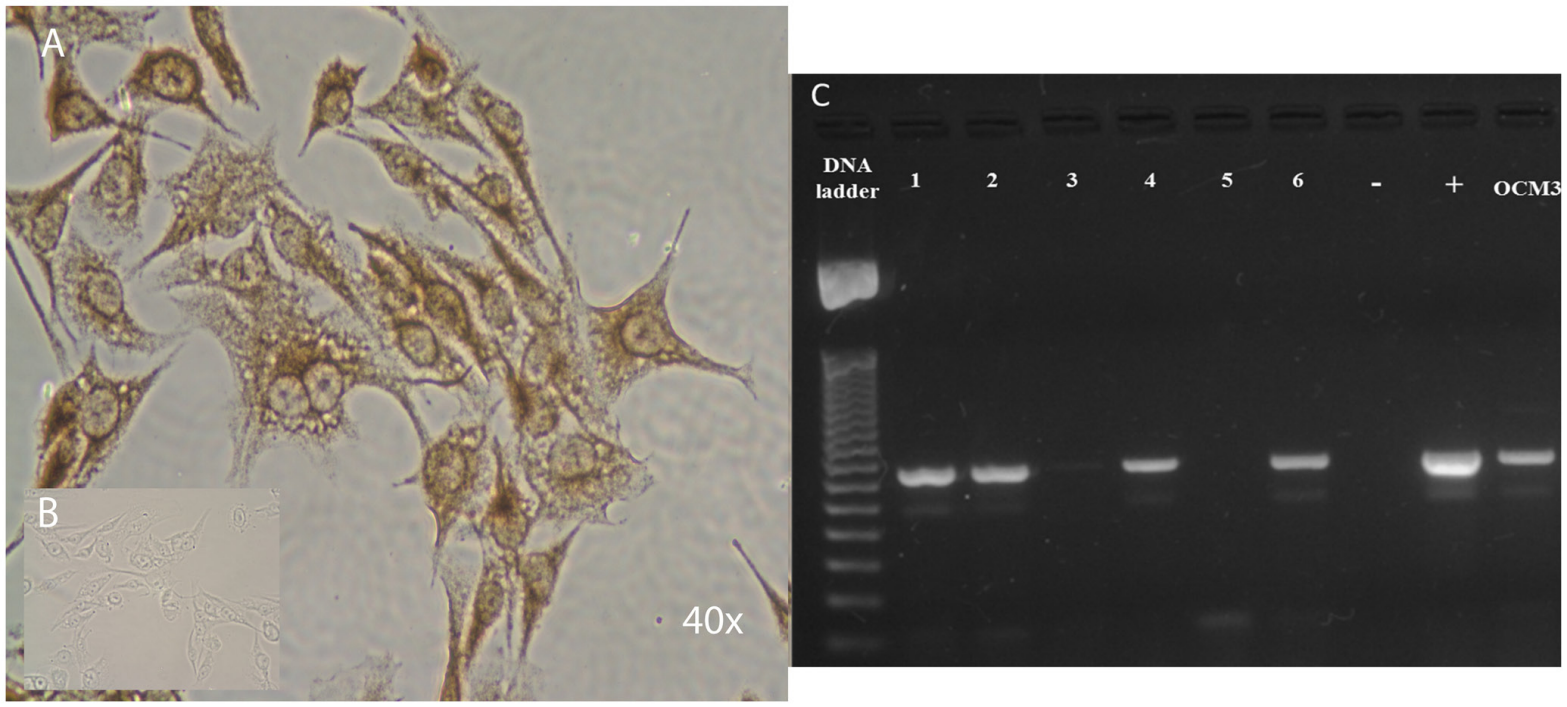
(**A**) Strong positivity of the full length LHRH receptor as detected by specific antibody in the nucleus, in the cytoplasm and in the membrane by DAB-immunoperoxidase staining. (**B**) Insert is a negative control for the staining-specificity. Original magnifications of images in immuncitochemistry: 40 ×. (**C**) Expression of LHRH receptor type I in OCM3 human uveal melanoma cells. The expected PCR products amplified with gene specific primers with 319 bp were detected successfully in OCM3 cell line. DNA ladder: 50 bp ladder (Fermentas), +: positive control (human pituitary); –: no template control, No. 1–6: representative human uveal melanoma tissues.

Expression and cellular distribution of the full length LHRH receptor type I in OCM3 cells was demonstrated by RT-PCR and by immunocytochemistry (Figure 1). The expected 319 bp PCR product, amplified with gene specific primers, was detected successfully in OCM3 cell line. Our result was confirmed also at protein levels by immunocytochemistry. We found that full length LHRH receptors are present in the cell membrane and therefore in the cytoplasm, so they might play a role in the facilitation of the selective uptake of AEZS-108 in OCM3 cells.

### AEZS-108 and doxorubicin induces comparable cytotoxicity in OCM3 cells

**Figure 2:**
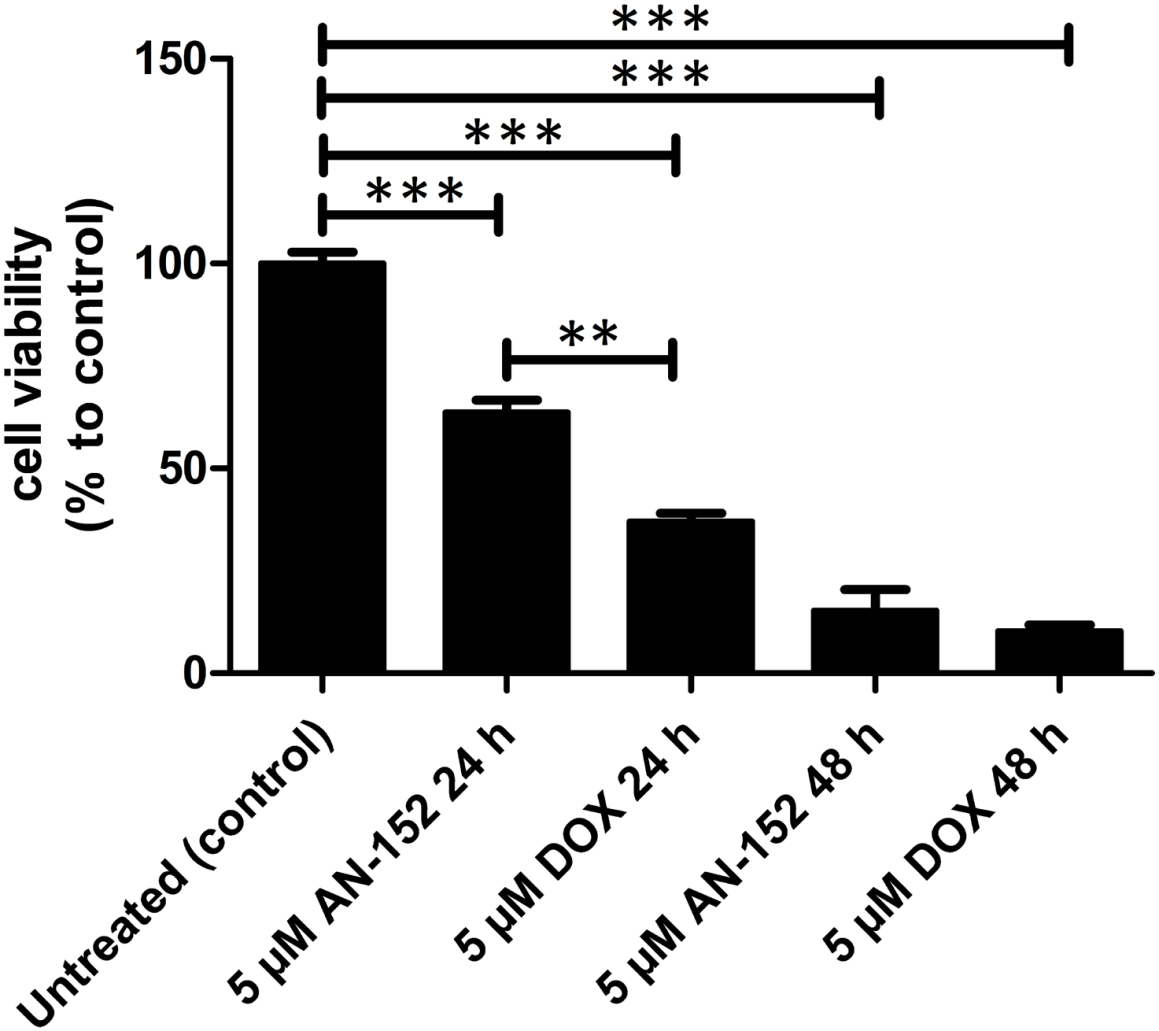
The cytotoxicity of AEZS-108 and DOX in OCM3 cells. The effect of treatment with 5 µM AEZS-108 and 5 µM DOX for 24 and 48 hours on cell viability of OCM3 cells was measured by MTS assay in complete medium. Statistical analysis was performed by one-way ANOVA (^**^: highly significant, *p* < 0.01; ^***^: extremely significant, *p* < 0.005).

In order to investigate whether AEZS-108 inhibits cell proliferation and its extent, OCM3 cells were treated either with 5 µM AEZS-108 or equal amount of doxorubicin. MTS assay was performed after 24 and 48 hours of treatment. AEZS-108 and doxorubicin have been shown to reduce cell proliferation by 36.3% (*p* < 0.001) and 62.9% (*p* < 0.001) respectively after 24 hours, and by 84.7% (*p* < 0.001) and 89.7% (*p* < 0.001) respectively after 48 hours, (Figure 2).

### AEZS-108 alters the expression of angiogenesis and metastatis regulatory factors in OCM3 cells

We have investigated the role of AEZS-108 in the expression of 94 key regulatory genes involved in angiogenesis and development of metastasis in OCM3 cells. *MASPIN (SERPINB5), SERPINE1 (PAI-1), CXCR4, VEGFA* and *MAPK7* genes have been found to be significantly upregulated, while *ANGPT1, HIF1A, ANGPTL3, ETS1, VEGFB, CEACAM* and *SURVIVIN* genes were significantly downregulated (*p* < 0.05) as compared to control (untreated) cells. The tumor suppressor gene *MASPIN (SERPINB5)* showed the highest overexpression (203.19× upregulation), while the most significantly downregulated gene was *HIF1A* (8.67× downregulation) (Tables 1 and 2).

**Table 1:** *In vitro* results of significantly upregulated genes following 5 µM AEZS-108 treatment in OCM3 cells

Symbol of gene	Upregulation (treated/control)	*p* value
*MASPIN (SERPINB5)*	+203.19	0.0021
*SERPINE1(PAI1)*	+52.59	0.0109
*CXCR4*	+15.45	0.0052
*VEGFA*	+3.48	0.0005
*MAPK7*	+2.27	0.0354

Results were normalized by the Global Pattern Recognition algorithm (Bar Harbor BioTechnology Inc.’s, USA), and quantified by ∆∆Ct method. Differences between the expression of genes between groups were tested for statistical significance level using Student’s *t*-test. (significant: *p* < 0.05).

**Table 2:** *In vitro* results of significantly downregulated genes following treatment with 5 µM AEZS-108 in OCM3 cells

Symbol of gene	Downregulation (treated/control)	*p* value
*ANGPT1*	–32.00	0.0220
*HIF1A*	–8.67	0.0187
*ANGPTL3*	–6.57	0.0433
*ETS1*	–5.66	0.0036
*VEGFB*	–5.10	0.0230
*CEACAM1*	–4.14	0.0082
*SURVIVIN*	–3.91	0.0007

Results were normalized by the Global Pattern Recognition algorithm (Bar Harbor BioTechnology Inc.’s, USA), and quantified by ∆∆Ct method. Differences between the expression of genes between groups were tested for statistical significance level using Student’s *t*-test. (significant: *p* < 0.05).

### AEZS-108 provokes higher expression of MASPIN than free DOX

**Figure 3:**
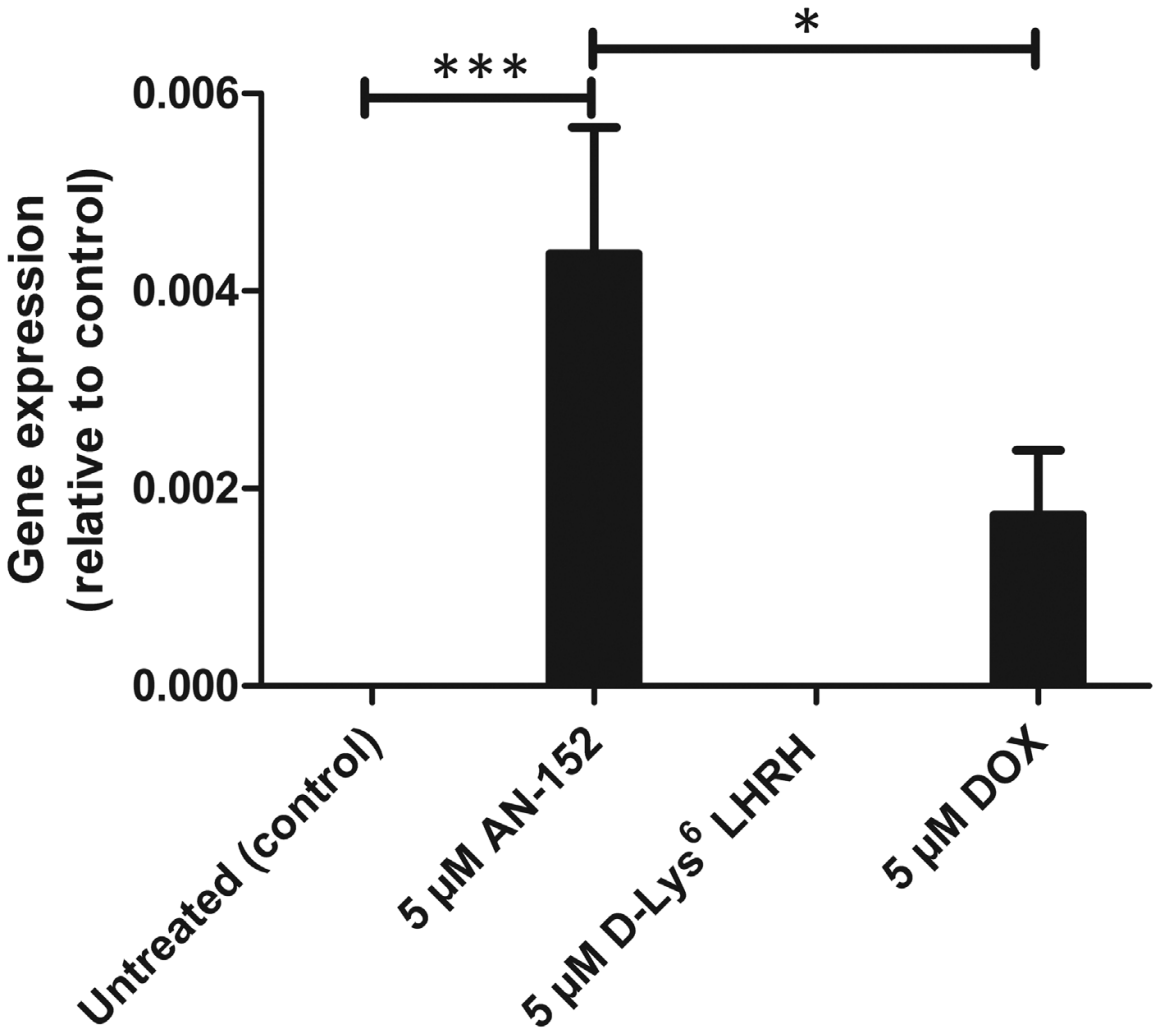
Treatment with AEZS-108 at equal doses with DOX was significantly more effective in the upregulation of *MASPIN* than free DOX. The expression of *MASPIN* tumor suppressor was measured by qRT-PCR from three independent experiments. For comparison, the corresponding mRNA expression levels of *MASPIN* are shown below the qPCR graphs for each treatments. Statistical analysis was performed by one-way ANOVA (^*^: significant, *p* < 0.05; ^***^: extremely significant, *p* < 0.005).

The expression of *MASPIN* tumor suppressor gene was further investigated by qRT-PCR. OCM3 cells were treated with AEZS-108, D-Lys^6^ LHRH analog or free DOX. Our results clearly showed that D-Lys^6^ LHRH-treated cells do not express *MASPIN*, while free DOX and AEZS-108 induces *MASPIN* expression. However, equal dose of AEZS-108 and DOX show significantly different effect on *MASPIN* expression, namely, that AEZS-108 treatment results in significantly higher *MASPIN* expression than free DOX treatment (Figure 3).

### The tumor suppressor MASPIN is not expressed in uveal melanoma or in normal uvea tissue specimens

The mRNA expression of *MASPIN* has been investigated in 3 healthy and 18 human uveal melanoma tissue specimens by real time PCR. Our results did not show mRNA expression of *MASPIN* in healthy or in uveal melanoma tissues (data not shown).

### AEZS-108 alters MASPIN, HIF1A, VEGFA and VEGFB protein expression in OCM3 cells

**Figure 4:**
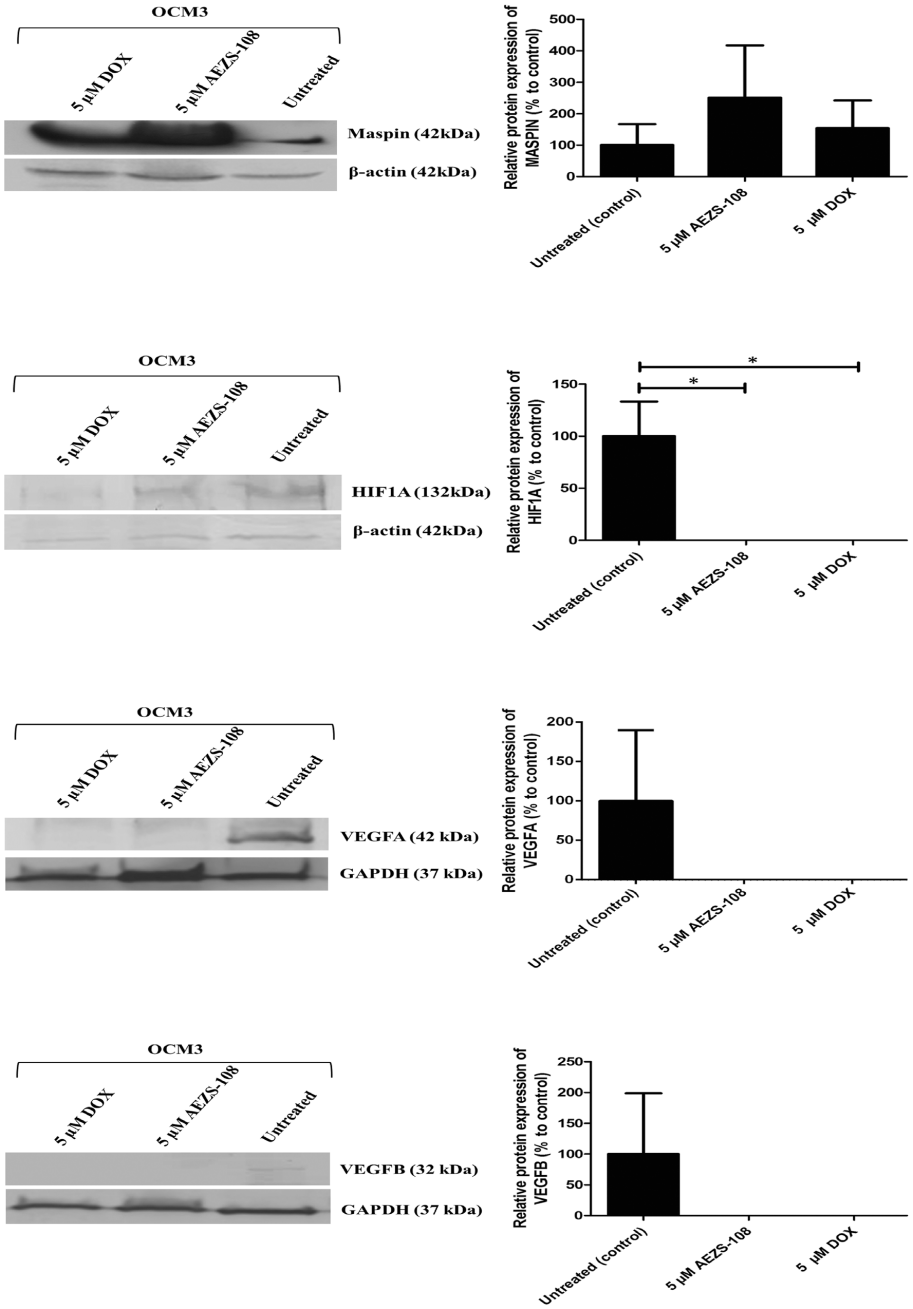
Western blot analysis of MASPIN, HIF1A, VEGFA and VEGFB protein expression after 24 hours of treatment with 5 µM AEZS-108 and 5 µM free DOX treatments from OCM3 cells. 40 µg of protein from each sample was separated on 12% SDS PAGE and transferred to PVDF or nitrocellulose membranes and probed with specific antibodies (described in Materials and metods). Data represents the densitometric analysis of the results to the target proteins normalized to β-actin or GAPDH. Statistical analysis was performed by one-way ANOVA (^*^: significant, *p* < 0.05).

Protein levels of MASPIN, HIF1A, VEGFA and VEGFB have also been examined. SDS PAGE Western blot analysis confirmed the qRT-PCR results, namely, that MASPIN production in untreated OCM3 cells is very low, however, treatment with AEZS-108 or free DOX slightly increases the expression of MASPIN tumor suppressor (Figure 4). As seen at mRNA and protein levels, AEZS-108 is a more potent inducer of MASPIN than free DOX. Furthermore, in order to unravel whether AEZS-108 or free DOX has an effect on angiogenesis, HIF1A and its target proteins, VEGFA and VEGFB angiogenesis related proteins were also investigated. Our data showed that treatment with AEZS-108 and DOX significantly decreased HIF1A, VEGFA and VEGFB expression (Figure 4).

## DISCUSSION

Although uveal melanoma is very rare, it is the most common primary intraocular malignant cancer and the second most common primary malignant melanoma in humans [4]. Death usually occurs within a year of the onset of systemic symptoms [3, 4]. The liver is a virtual “sentinel lymph node” for uveal melanoma since unfortunately it is involved in 95% of patients with metastasis [4]. The absence of effective therapy for metastatic disease offers now a chessboard for targeted therapy, creating new opportunities to develop novel treatment options [36–40].

It has been shown that the growth of various cancer cell lines, e. g. cutaneous melanoma xenografted into nude mice can be inhibited by analogs of targeted cytotoxic luteinizing-hormone-releasing hormone (LHRH) [41]. Interestingly, our previous results showed that 46% of specimens of uveal melanoma tissues express LHRH receptor type I [1]. Previous studies have demonstrated that AEZS-108 strongly inhibits the growth of experimental human prostatic, mammary, ovarian and urinary bladder cancers as well as melanomas expressing LHRH receptors [6, 12, 27, 30, 32, 33, 41, 42]. Previous *in vivo* investigations have demonstrated that AEZS-108 has a prominent antitumor activity and less toxicity than unconjugated DOX in various cancer types [7–9, 11–13, 43]. Upon detection of LHRH receptors in specimens of human uveal melanoma, we have investigated whether AEZS-108, which is appropriate for targeted therapy of various tumors, could serve as a novel therapy in UM.

Our aim was to further elucidate the antitumor effects of AEZS-108 in human UM. As a relevant *in vitro* model of UM, OCM3 cell line was selected in order to investigate the antitumor-effect of AEZS-108 compared to its unconjugated cytotoxic consituent, doxorubicin. First, we examined the expression of LHRH receptors in OCM3 cells by qRT-PCR and by immunocytochemistry. To demonstrate if AEZS-108 induces cytotoxicity, OCM3 cells were treated with 5 µM AEZS-108 or equal amount of doxorubicin and then MTS-assay was performed. Our result show that AEZS-108 reduces cell viability by 36.3% after 24 hours (*p* < 0.001) and by 84.7% after 48 hours (*p* < 0.001). In contrast, free DOX led to 62.9% (*p* < 0.001) and 89.7% (*p* < 0.001) cell death after 24 and 48 hours, respectively. These results might be explained by the fact, established previously, that some cytotoxic ligand analogs containing DOX show ten times lower antiproliferative activity *in vitro* than doxorubicin. However, *in vivo* experiments are in accord with the *in vitro* results and demonstrate that cytotoxic ligand analogs present high antitumor activity, greater efficacy and less toxicity than doxorubicin [7–9, 32].

Since lymphatic vessels are absent in the eye, angiogenesis plays a pivotal role in the development of UM and subsequently, in the development of metastasis [35, 36, 44, 45]. Based on the special characteristics of uveal melanoma, our further aim was to investigate the effect of AEZS-108 on the expression of the genes related to angiogenesis and migration in OCM3 cells by qRT-PCR array. The tumor suppressor *MASPIN (SERPINB5)* and *SERPINE1* genes showed the highest overexpression, while the most significantly downregulated genes were *HIF1A* and its target genes, also involved in angiogenesis and metastasis of cancer cells. The observed changes in mRNA expression have been confirmed at protein level by Western blot analysis.

In terms of function these findings support a central role for *MASPIN* and *HIF1A* in ECM and in the regulation of angiogenesis in UM whereas the published data on role of these two genes for the survival of UM is relatively limited [46, 47].

**Figure 5:**
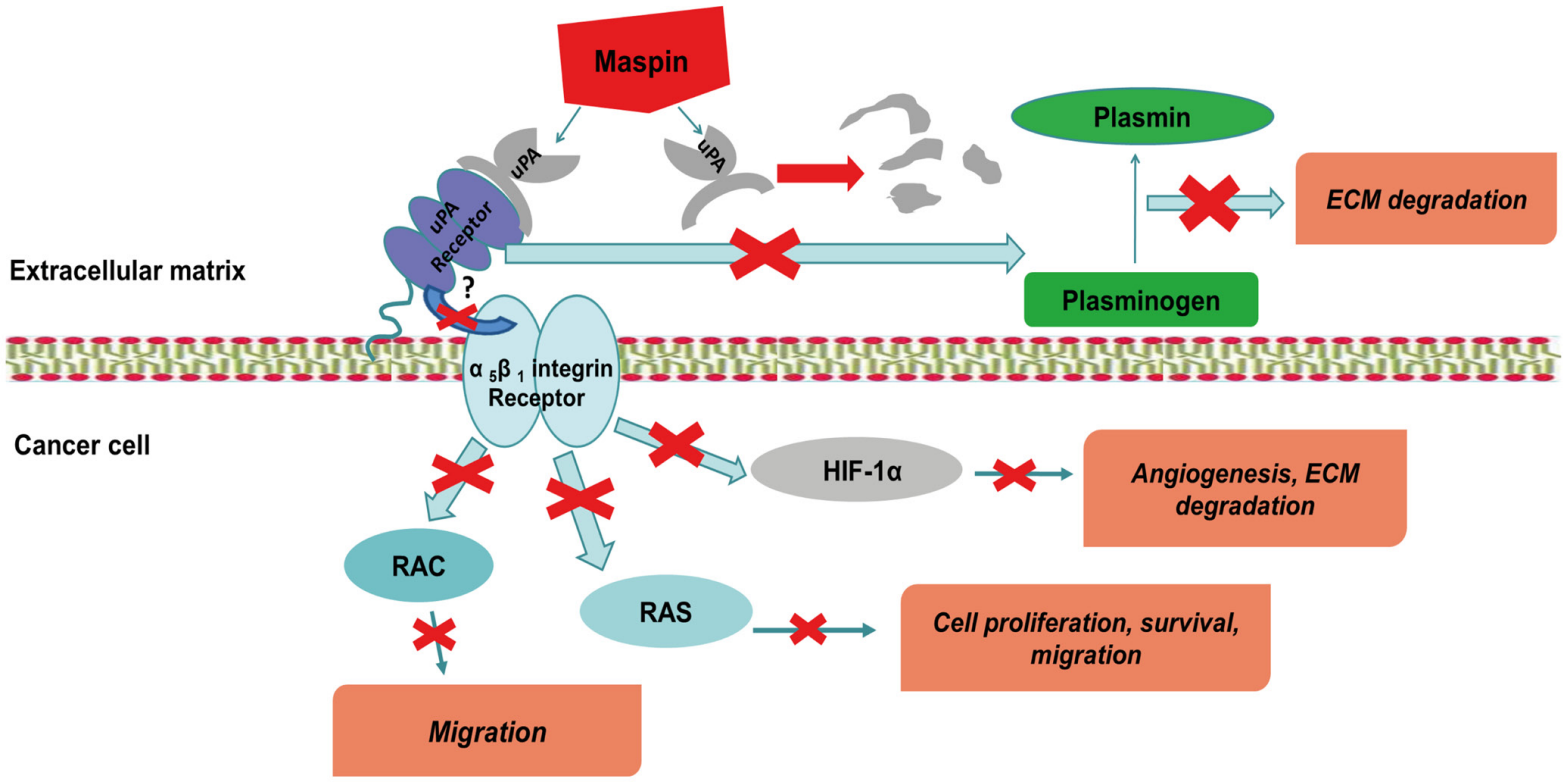
MASPIN can prevent the formation of UPA - UPA-receptor complex by a single step, and thus decrease the possibility of the abnormal degradation of the ECM, the development metastasis and angiogenesis. (UPA: urokinase type plasminogen activator, UPAR: UPA-receptor, HIF1A: hypoxia-inducible factor 1 alpha ECM: extracellular matrix, RAS: GTP-binding protein, RAC: subfamily of RHO-GTP-ases).

Previous studies have demonstrated the role of *MASPIN* and *SERPINE1* in the coordination of ECM proteolytic system and its important functions in cell migration, proliferation and survival [46]. *MASPIN* and *SERPINE1* are natural inhibitors of *uPA/uPAR* signaling, which is strengthened during inflammation, tissue remodeling and in many cancers, frequently indicating a poor prognosis [46] (Figure 5). We have detected that both *MASPIN* and *SERPINE1*, *uPAR* signaling inhibitors were simultaneously upregulated by treatment with AEZS-108. Controversially, *HIF1A*, the central regulator of angiogenesis was downregulated. *HIF1A* induces tumor hypoxia driving *uPAR* expression through a *hypoxia responsible element* (*HRE*) in the *uPAR* promoter. Furthermore, *uPAR* is required for the induction of an epithelial-mesenchymal transition-like response in other cancer cell lines following exposure to hypoxia [46, 48]. Previously demonstrated data in accordance with our results might suggest that AEZS-108 induces a well regulated signaling pathway against ECM degradation, angiogenesis and migration of tumor cells via the regulation of *MASPIN*, *HIF1A* and their target genes [46].

In order to investigate the mechanism of the induction of *MASPIN*, OCM3 cells were treated with free DOX, D-Lys^6^ LHRH analog, or AEZS-108. AEZS-108 induced greater *MASPIN* expression compared to free DOX. Unconjugated D-Lys^6^ LHRH analog had no effect on the induction of *MASPIN*. Based on our results, we assume that the induction of *MASPIN* is independent of the LHRH ligand-receptor interaction, and is probably triggered only by the cytotoxic DOX unit. The upregulation of *MASPIN* expression might be explained by a probable decrease in transporter activity in the presence of AEZS-108 [49].

We have also evaluated the expression of *MASPIN* expression in healthy human uvea and in human UM tissue specimens. According to our results, *MASPIN* is undetectable in healthy uvea and in UM tissues. Diminished expression of *MASPIN* in the UM specimens is congruent with a previous study, in which the decreased *MASPIN* expression in UM tissue specimens indicated poor prognosis due to therapy resistance [50].

In summary, our data confirmed previous results showing LHRH receptor expression in OCM3 cells, an UM *in vitro* model. Furthermore, we report for the first time that AEZS-108 causes changes in the expression of genes which are involved in angiogenesis and ECM degradation and which might inhibit cell proliferation and induce apoptosis in OCM3 cells.

These findings suggest that AEZS-108 plays a pivotal role in the regulation of angiogenesis and tumor suppression. Taken together, targeted cytotoxic LHRH analogs, such as AEZS-108, might serve as an effective treatment for patients with LHRH receptor positive uveal melanoma.

## MATERIALS AND METHODS

### Ethics statement

The local Institutional Ethics Committee of the University of Debrecen approved the collection and use of human pituitary, normal uvea and uveal melanoma specimens for the current study and informed consent was obtained from these patients (ID number: DERKEB/IKEB 4172-2014). Uvea and uveal melanoma specimens were obtained at the time of initial surgical treatment at the Department of Ophthalmology, University of Debrecen, Hungary. Normal pitutaries were collected by autopsy at the Department of Pathology, University of Debrecen, Hungary.

### Cell line and culturing conditions

The human metastatic uveal melanoma cell line, OCM3 (ocular choroideal melanoma 3, cultured from primary tumor) was kindly provided by the Department of Biophysics and Cell Biology, Faculty of Medicine, University of Debrecen. OCM3 cells were cultured in complete growth medium: RPMI 1640 medium supplemented with L-glutamine (PPA Labotatories, Austria), 10% fetal bovine serum (FBS) (PPA Labotatories, Austria) and 100 U/ml Penicillin, 100 mg/ml Streptomycin (Pen/Strep, TEVA Pharmaceuticals Zrt., Hungary) in a humidified chamber (95% air humidity, 5% CO_2_) at 37°C.

### Detection of LHRH receptor in OCM3 cells by immunocytochemistry

Cells were seeded into a 96-well plate at the density of 25.000 cells/well in complete growth medium and were left to adhere overnight. On the following day, cells were rinsed with PBS (pH 7.4) and fixed in cold methanol for 10 minutes. Endogenous peroxidase activity was blocked with 3% H_2_O_2_. Cells were permeabilized by adding 0.1% Triton X-100 in PBS. Non-specific binding sites were blocked with 1% FBS and 0.1% Triton X-100 containing PBS (60 min, room temperature). Cells were incubated with human LHRH receptor type I antibody (Santa Cruz Biotechnology, USA, FL-328; sc-13944) in 1:100 dilution in PBS overnight at 4°C. Next day, cells were stained with horseradish peroxidase (HRP)-conjugated secondary antibody (anti-rabbit IgG (Fab) of EnVision+ kit, (DM822) Dako, Denmark) according to the manufacturer’s instructions. Human pituitary glands (anterior lobe) obtained from autopsy at the University of Debrecen were used as positive controls.

Negative controls (primary antibody was replaced by normal serum) were also included. DAB chromogenic staining of the receptors was detected by SPOT^™^ Imaging Software (Dioganostic Instruments Inc., USA) and Nikon Eclipse TS/100 microscope (Nikon Instruments, Melville, NY).

### Detection of LHRH receptor type I in OCM3 cells after RNA isolation by reverse transcription (RT)-PCR

Total RNA was isolated from OCM3 cells using the AllPrep DNA/RNA/Protein Mini kit (Qiagen, Germany). 500 ng of RNA from each sample was reverse transcribed into cDNA by QuantiTect Reverse Transcription kit (Qiagen, Germany). The following primer pair has been used to detect LHRH-R type I: 5′- GAC CTT GTC TGG AAA GAT CC -3′ (sense), 5′- CAG GCT GAT CAC CAC CAT CA -3′ (antisense) (Sigma-Aldrich Corporation, USA). Primers were designed using Primer3web softwer. One μl of cDNA was amplified in a 25 μl solution containing 1.5 mM MgCl_2_, 1× PCR buffer (Fermentas, Germany), 0.3 mM of each deoxynucleotide (Promega, Germany), 1 unit of TrueStart HotStart DNA polymerase (Fermentas, Germany) and 0.25 μM of each primer. Samples were subjected to 40 cycles of 95°C for 45 s, 59°C for 30 s, then 72°C for 1.5 min with a final extension of 10 min at 72°C. Normal human pituitaries, collected at autopsy, served as positive control. Human uveal melanoma specimens, previously investigated by us [1] were also used to confirm the expression of LHRH receptors. No template control (NTC) was used to detect potential contaminations in the RT-PCR reactions. PCR cycles have been performed using the Bio-Rad C1000^™^ Thermal Cycler (Bio-Rad Laboratories, USA) instrument. 10 μl of each amplification reaction was then electrophoretically separated on 1.5% agarose gel, stained with GelRed (Biotinum, USA), and visualized under UV light.

### Cytotoxic and LHRH ligand analogs and doxorubicin

The targeted cytotoxic analog, AEZS-108 (formerly known as AN-152/ INN: Zoptarelin Doxorubicin Acetate), corresponding to D-Lys^6^ LHRH (pyroGlu-His-Trp-Ser-Tyr-D-Lys-Leu-Arg-Pro-Gly-NH2) linked to doxorubicin (DOX) was first sythesized by solid-phase method, as described by the laboratory of Andrew V. Schally [6, 7, 42]. The AEZS-108 and D-Lys^6^ LHRH used in our experiments was kindly provided by AEterna/ Zentaris (Frankfurt am Main, Germany). Doxorubicin-HCl (2 mg/ml) (DOX) was purchased from Teva Pharmaceutical Works Ltd. (Hungary).

The compounds were dissolved in NaCl (Salsol-A, Teva Pharmaceutical Works Ltd., Hungary) containing 0.01 M aqueous acetic acid at a stock concentration of 100 µM. Based on previous studies, compounds were used in 5 µM concentration [33, 51].

### Determination of cell viability by MTS assay

Cells were seeded into a 96-well plate at the density of 10.000 cells/well in complete growth medium and incubated for 24 hours. Afterwards, medium was completely replaced with 5 µM cytotoxic compound-containing growth medium and cells were incubated for additional 24 hours. Subsequently, MTS assay was performed in order to quantify viable cells (CellTiter 96 AQueous One Solution Assay Assay (Promega, Madison, WI). The assay was done according to the manufacturer’s protocol. All treatments were performed in hexaplicates (*n* = 6). Absorbance was measured at 490 nm in a FLUOstar Optima Counter (BMG Labtech GmbH, Germany). Values were expressed relative to the control.

### Treatment of OCM3 cells and RNA isolation

OCM3 cells were seeded and cultured in T25 cell culture flasks for 24 hours, until forming a confluent monolayer. Next day the medium was discarded and replaced with either 5 µM AEZS-108, DOX or D-Lys^6^ LH-RH analog containing complete growth medium. Cells were incubated for additional 24 hours. The treatment was performed in biological replicates (*n* = 3). Total RNA was isolated from treated and untreated (control) OCM3 cells by TRI Reagent (MRC, USA) according to the manufacturer’s instructions. The homogenization of the tissue samples was performed with TissueRuptor^®^ (Qiagen, Germany). RNA isolation process were carried out as previously described [52].

### RT- PCR and qRT-PCR with OCM3 samples

Gene expression changes induced by treatment in the OCM3 cells were evaluated after RT-PCR. 500 ng of total RNA from each sample was transcribed into cDNA IScript Reverse Transcriptase Kit (Bio-Rad Laboratories, USA) respectively, according to the manufacturer’s instructions.

In order to investigate the expression of genes involved in angiogenesis and metastasis regulatory factors after treatment of OCM3 cells with cytotoxic AEZS-108, Human Angiogenesis 96 StellArray^™^ (Lonza Ltd., USA) plates and SYBR Green Supermix (Bio-Rad Laboratories, USA) were used according to the manufacturer’s protocol. qRT-PCR arrays were performed with untreated and AEZS-108- treated OCM3 samples in biological replicates (*n* = 3). Samples were subjected to an initial anellation step (50°C for 2 min) followed by the activation of enzyme (95°C for 3 min), then 40 cycles of amplification (95°C for 15 sec, 60°C for 1 min) and 81 cycles of melting curves (55°C – 98°C for 0.5 min/°C). Results were normalized by the Global Pattern Recognition algorithm (Bar Harbor Bio Technology Inc.’s, USA) and quantified by ΔΔCt method.

Based on the results of the array, particularly, the expression of *MASPIN* tumor suppressor gene has been further investigated after treatment with AEZS-108, DOX or D-Lys^6^ LHRH using SYBR Green Supermix (Bio-Rad Laboratories, USA) in a 20 µl total volume according to the manufacturer’s instructions. The following primer pair has been used for *MASPIN*: 5′ -TCAACAAGACAGACACCAAACC - 3′ (sense), 5′- GGAACTCATCCTCCACATCCT - 3′ (Sigma-Aldrich Corporation, USA). Primers were designed using Primer3web softwer. PCR cycles were run as described in the qRT-PCR array experiment. Gene expression has been quantified by the 40- C_T_ method. Results were normalized to the expression of β-actin and HPRT housekeeping genes. cDNA sample from HaCaT cells was used as a positive control and no template control (NTC) to detect potential contaminations in the reactions. PCR cycles have been performed using the Bio-Rad C1000^™^ Thermal Cycler and the Bio-Rad MyiQ2^™^ Real Time qPCR (Bio-Rad Laboratories, USA) instruments.

### RNA isolation and reverse transciption of human tissue samples

Human uveal melanoma specimens were obtained from 18 patients, 30–84 years old at the time of enucleation. Three normal uvea samples were collected from healthy patients after severe eye accident. After surgical removal, selected portions of the tissues were snap-frozen and stored at –70°C. In all cases, histopathological examination confirmed the diagnosis. Total RNA was isolated using the AllPrep DNA/RNA/Protein Mini kit (Qiagen, Germany). 500 ng RNA from each sample was reverse transcribed into cDNA by QuantiTect Reverse Transcription kit (Qiagen, Germany) according to the manufacturer’s instructions.

### Detection of MASPIN with RT-PCR in human uveal melanoma and healthy uvea tissue specimens

Based on our qRT-PCR results, the gene expression of *MASPIN* (SERPINB5) has been further examined in human uveal melanoma and healthy human uvea specimens. The following primer pairs were used: 5′ - GGCAATGTCCTCTTCTCTCC - 3′ (sense), 5′- GCCGCTTGATTAGTTTCAGT - 3′ (antisense) (Sigma-Aldrich Corporation, USA). Primers were designed using Primer3web softwer. 1 μl of the cDNA was amplified in 25 μl solution containing 1.5 mM MgCl_2_, 1xPCR buffer (Fermentas, Germany), 0.3 mM of each deoxynucleotide (Promega, Germany), 1 unit of DNA polymerase (Fermentas, Germany) and 0.1 μM of each primer. Samples were subjected to an initial denaturation (95°C for 3 min) followed by 35 cycles of amplification (95°C for 45 sec, 60°C for 30 sec, 72°C for 1.5 min) and a final elongation step (72°C for 10 min). As positive control we used HaCaT cDNA samples. No template control (NTC) has been used to detect potential contaminations in the reactions. 10 μl of each amplification reaction was then electrophoretically separated on 1.5% agarose gel, stained with GelRed (Biotinum, USA), and visualized under UV light.

### Protein analysis

Total protein was isolated from untreated, AEZS-108- or DOX-treated cells using TRI Reagent (MRC, USA) and purified with isopropyl alcohol (Sigma-Aldrich Corporation, USA), 300 mM guanidine-hydrochloride (Amresco, VWR) and 96% ethanol (Scharlab, Hungary) according to the manufacturer’s instructions. The final pellet was resuspended in 1% SDS buffer containing protease inhibitors; 0.4% (v/v) aprotinin (Sigma-Aldrich Corporation, USA), 2 μg/ml pepstatin A (Sigma-Aldrich Corporation, USA) and leupeptin (Sigma-Aldrich Corporation, USA) and stored until use at –80°C. Protein concentration was determined by Bradford method [53, 54]. 40 µg protein has been separated on 12% SDS PAGE and transferred to PVDF or nitrocellulose membrane. 5% nonfat dry milk or BSA has been used as blocking agent in Tris buffered saline containing 0.1% Tween-20. Primary antibodies (Santa Cruz Biotechnology, USA and Cell Signaling Technology, USA) were applied at the following concentrations: MASPIN (C-8; sc-271694) in 1:100; HIF1A (HIF1A (H-206; sc-10790) in 1:200; VEGFA (VEGF (A-20; sc-152) in 1:100; VEGFB (VEGFB (MM0008-7B43; sc-101581) in 1:200 and GAPDH (D16H11 XP^(R)^) in 1:1000. Primary antibodies were stained with alkaline-phosphatase or horseradish-peroxidase conjugated secondary antibodies (Santa Cruz Biotechnology, USA) and detected using AP Conjugate Substrate Kit (Bio-Rad Laboratories, USA) or WesternBright^™^ ECL Substrate Kit (Advanstra Corporation, USA). Results were acquired and analyzed with the Molecular Image Chemidoc XRS+ System using Image Lab Software 5.2 (Bio-Rad Laboratories, USA).

### Statistical analysis

Data were analyzed by Students’s *t*-test or one-way ANOVA followed by Tukey post hoc test to determine the differences between the selected groups using the program Prism 5 software (GraphPad Software, Inc., USA) and with IBM SPSS Statistics (IBM Corp. Released 2014. IBM SPSS Statistics for Windows, Version 23.0. Armonk, NY: IBM Corp.). Data are presented as a mean value ± standard error of the mean (SEM). A *p* value ^*^<0.05 was considered to be statistically significant (*p* value ^**^<0.01: highly significant, *p* value ^***^<0.005: extremely significant).

## Abbreviations

UM: uveal melanoma
OCM3: ocular chorioideal melanoma 3, cultured from primary tumor
LHRH: Luteinizing Hormone-Releasing Hormone
LHRH-R: Luteinizing Hormone-Releasing Hormone receptor
DOX: doxorubicin
D-Lys^6^ LHRH analog: (pyroGlu-His-Trp-Ser-Tyr-D-Lys-Leu-Arg-Pro-Gly-NH2) LHRH analog
AEZS-108 (formerly known as AN-152/INN: Zoptarelin Doxorubicin Acetate): D-Lys^6^ LHRH analog linked to doxorubicin
*HIF1A*: Hypoxia-inducible factor 1 alpha
*MASPIN (SERPINB5)*: Serpin peptidase inhibitor clade B, member 5
*SERPINE1 (PAI-1)*: Serpin family E, member 1
*ETS1*: ETS Proto-Oncogene 1, Transcription Factor
*CEACAM*: Carcinoembryonic Antigen Related Cell Adhesion Molecule
*SURVIVIN*: BIRC5, Baculoviral IAP Repeat Containing 5
*VEGFA*: Vascular Endothelial Growth Factor A
*VEGFB*: Vascular Endothelial Growth Factor B
*ANGPT1*: Angiopoietin 1
MAPK7: Mitogen-Activated Protein Kinase 7
CXCR4: C-X-C Motif Chemokine Receptor 4
ECM: extracellular matrix
qRT- PCR: quantitative reverse transcription- polymerase chain reaction
FBS: fetal bovine serum
PBS: phosphate buffered salin
HRP: horseradish peroxidase
